# Selective regulation of YB-1 mRNA translation by the mTOR signaling pathway is not mediated by 4E-binding protein

**DOI:** 10.1038/srep22502

**Published:** 2016-03-02

**Authors:** D. N. Lyabin, L. P. Ovchinnikov

**Affiliations:** 1 Institute of Protein Research, Russian Academy of Sciences, Pushchino, 142290, Russian Federation

## Abstract

The Y-box binding protein 1 (YB-1) is a key regulator of gene expression at the level of both translation and transcription. The mode of its action on cellular events depends on its subcellular distribution and the amount in the cell. So far, the regulatory mechanisms of YB-1 synthesis have not been adequately studied. Our previous finding was that selective inhibition of *YB-1* mRNA translation was caused by suppression of activity of the mTOR signaling pathway. It was suggested that this event may be mediated by phosphorylation of the 4E-binding protein (4E-BP). Here, we report that 4E-BP alone can only slightly inhibit YB-1 synthesis both in the cell and *in vitro*, although it essentially decreases binding of the 4F-group translation initiation factors to mRNA. With inhibited mTOR kinase, the level of mRNA binding to the eIF4F-group factors was decreased, while that to 4E-BP1 was increased, as was observed for both mTOR kinase-sensitive mRNAs and those showing low sensitivity. This suggests that selective inhibition of translation of *YB-1* mRNA, and probably some other mRNAs as well, by mTOR kinase inhibitors is not mediated by the action of the 4E-binding protein upon functions of the 4F-group translation initiation factors.

YB-1 is a multifunctional DNA- and RNA-binding protein belonging to the family of proteins with an evolutionary conserved cold shock domain. YB-1 participates in many cellular events and is involved in virtually all stages of transfer and realization of the genetic information, specifically, transcription and mRNA translation^1^,^2^. However, the knowledge of regulation of YB-1 synthesis is still insufficient.

As we showed previously, YB-1 synthesis depends on the rate of cell division. Suppression of cell division resulted in selective inhibition of YB-1 synthesis in the cell, while its recovery caused a rapid and specific increase in YB-1 synthesis^3^. Our supposition was that this phenomenon is underlain by sensitivity of *YB-1* mRNA translation to activity of the mTOR signaling pathway that regulates cell growth and cell response to various growth stimuli, stresses, and availability of nutrients and energy^4^. A key member of the signaling pathway is mTOR kinase, a constituent of the complexes mTORC1 and mTORC2. It is agreed that mTORC1 is responsible for translation regulation. The best studied substrates of mTORС1 are p70S6 kinase and 4E-binding protein (4E-BP). The latter is believed to play the key role in translation inhibition through suppression of activity of the mTOR signaling pathway^5^,^6^. Its dephosphorylated form binds to eIF4E, thereby preventing interaction of this factor with other eIF4F-group factors (eIF4G and eIF4A) and their binding to mRNA^6^,^7^. Particular sensitivity to inhibition of the mTOR signaling pathway is displayed by mRNAs with the terminal olygopyrimidine tract (TOP) or a TOP-like sequence at their very 5′ end or within 5′ UTR (e.g., pyrimidine-rich translational element (PRTE)^5^), although the presence of the TOP motif is not always indispensable or sufficient^8^.

It was our previous finding that mTOR inhibition by selective inhibitors pp242 and Torin2, that suppress phosphorylation of 4E-BP and p70S6 kinase, caused in the cell specific inhibition of both YB-1 synthesis and translation of reporter mRNAs carrying the 5′ UTR of *YB-1* mRNA. However, rapamycin (another mTOR inhibitor affecting mostly p70S6 kinase) produced no effect on YB-1 synthesis^3^. This allowed supposition that mTOR-dependent regulation of YB-1 synthesis is mediated by phosphorylation of 4E-BP.

The current study is aimed to learn whether or not 4E-BP is the key protein in regulation of *YB-1* mRNA translation under mTOR inhibition. We showed that overexpression of mutant non-phosphorylatable 4E-BP1 in the cell or addition of recombinant 4E-BP1 to a cell-free translation system produced no noticeable effect either on synthesis of endogenous YB-1 in HeLa cells or translation of reporter mRNA carrying the *YB-1* mRNA 5′ UTR. Next, affinity chromatography on resin with immobilized capped mRNA fragments was used to show that, upon mTOR kinase inhibition, fragments of both mTOR sensitive and nonsensitive mRNAs demonstrated equally decreased binding to the factors eIF4E, eIF4G, and particularly eIF4A, with increasing binding to 4E-BP. Supposedly, specific inhibition of translation of *YB-1* mRNA and some other mRNAs occurring concurrently with inhibition of the mTOR signaling pathway is not explained by specifically changed affinity of the translation initiation factors for these mRNAs. Besides, according to our data, in the course of translation suppression with mTOR inhibitors, the crucial event is not only 4E-BP dephosphorylation preventing eIF4G-to-eIF4E binding on the mRNA but probably a lower level of mRNA binding to eIF4E and eIF4A, as well. This suggested a higher sensitivity of translation of *YB-1* mRNA and similar mRNAs to inhibition of cap-binding and RNA-helicase activities, which has been confirmed experimentally.

## Results

### The effect of mTOR inhibitor Torin2 and 4E-BP1 on translation of mRNAs carrying various 5′ UTR

**Figure 1:**
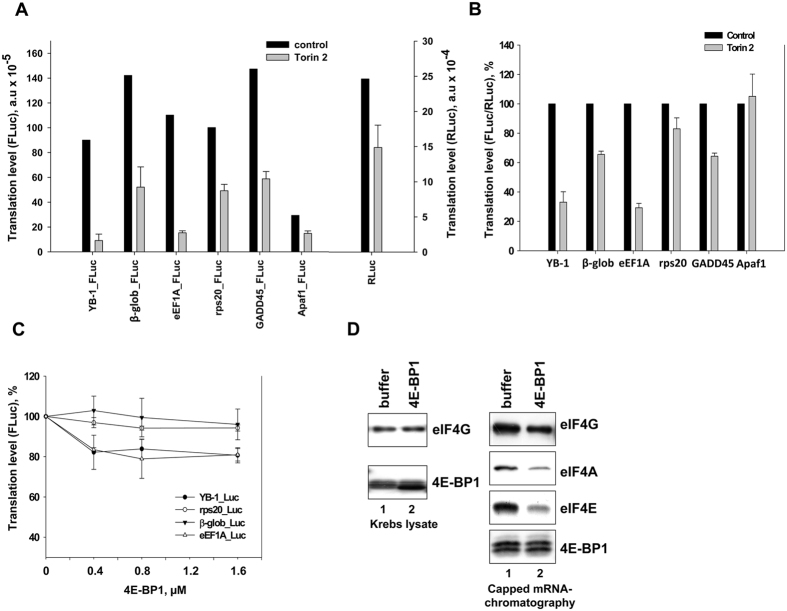
The effect of Torin2 on translation of reporter mRNAs in HeLa cells, and the effect of 4E-BP1 on translation of reporter mRNAs *in vitro*. (**A**) Untreated or Torin2-treated (0.25 μM, 1 h) HeLa cells were transfected by reporter *Firefly luciferase* mRNAs with indicated 5′ UTRs and *Renilla luciferase* mRNA (as internal control), cultivated for 2 h, harvested and assayed for *Firefly* and *Renilla luciferase* (*FLuc* and *RLuc*, respectively). Absolute values of *FLuc* or *RLuc* activities are presented. Errors are 2 standard deviations. (**B**) The same as (**A**) but the *FLuc*/*RLuc* ratio is presented. The *FLuc*/*RLuc* ratio for the control (untreated cells) was taken as 100%. Values are the means of at least three independent experiments. Errors are 2 standard deviations. (**C**), 0.1 pmol C^+^A^+^ reporter *Firefly luciferase* mRNAs with indicated 5′ UTRs were translated in Krebs extract in the presence of increasing amounts of recombinant 4E-BP1 (0.4, 0.8, and 1.6 pmol) or without it. Reaction mixtures were assayed for *Firefly* luciferase after 45 min incubation at 30 °C. The *FLuc* activity without addition of 4E-BP1 was taken to be 100%. Values are the means of at least three independent experiments. Errors are 2 standard deviations. (**D**) Translation reactions (Krebs extract) with 1.6 pmol 4E-BP1 or without it were analyzed by Western blotting (left panel) or used for analysis of binding of translation initiation factors. The biotinylated, capped *luciferase* mRNA with *β-globin* mRNA 5′ UTR (0.1 pmol) was incubated in 10 μl translation reaction mixture (Krebs extract) with or without 1.6 pmol 4E-BP1 and immobilized on Streptavidin-Sepharose. RNA-bound proteins were eluted, separated by SDS-PAGE, and analyzed by Western blotting (right panel).

As it was tentatively shown, in HeLa cells treated with mTOR kinase inhibitor Torin2, translation of reporter mRNAs with mTOR-sensitive 5′ UTRs from *YB-1*- and *eEF1A* mRNAs was strongly suppressed (~10-fold), in contrast to control mTOR-nonsensitive mRNAs with 5′ UTRs from *β-glob*-, *Apaf1*- and *GADD45* mRNAs (~1.5–2-fold) (Fig. 1A). It should be noted that in these experiments, *Renilla luciferase* mRNA carrying a short 15 nt 5′ UTR was used as a control (see Supplementary Fig. S1). Like any capped mRNA, this one was somewhat sensitive to mTOR inhibition, and showed suppression by about 1.6 times (Fig. 1A). Therefore, here, the FLuc/RLuc ratio (Fig. 1B) manifested the extent to which resistance to mTOR inhibition of this or that 5′ leader of the tested mRNA was increased or decreased, as compared with the control *RLuc* mRNA. For convenience, further on, the term “nonsensitive” will denote mRNAs that show low sensitivity to mTOR inhibition, while “sensitive” – those showing high sensitivity to it.

Translation of mRNAs carrying the *rps20* mRNA 5′ UTR was expected to be also strongly suppressed as a result of mTOR inhibition^6^, but no such suppression was observed in experiment. This could possibly be explained by the fact that all reporter mRNAs used in our experiments had some additional 12 nt sequence before their 5′ UTR (see Materials and Methods), which could affect translation of *rps20* mRNA because, as believed, TOP mRNAs (and *rps20* mRNA among them) require a terminal position of the TOP sequence at the 5′ end. However, it should be noted that the reporter mRNA carrying 5′ UTR of another TOP mRNA, namely, *eEF1A* mRNA, retained the degree of its sensitivity to mTOR inhibition. However, it cannot be ruled out that this degree would be below the one observed in the case of natural *eEF1A* mRNA 5′ UTR.

According to the literature, mRNA sensitivity for mTOR inhibition is 4E-BP-dependent^6^. Therefore, our experiments using HeLa cells and a cell-free translation system with Krebs extract were aimed to find whether 4E-BP1 alone is sufficient to inhibit translation of *YB-1* mRNA. As seen from Fig. 1C, addition of recombinant 4E-BP1 to Krebs extract resulted in a somewhat greater decrease in translation of the reporter mRNA carrying 5′ UTRs of *YB-1*- and *eEF1A* mRNAs, as compared to mTOR-nonsensitive mRNAs used as a control. The inhibitory effect was insufficient, and the translation level accounted for 70% of the initial value, as observed in contrast to mTOR inhibition-induced suppression of translation of mTOR-sensitive reporter mRNAs in HeLa cells.

It is seen in Fig. 1D that 4E-BP1 in its highest concentration (1D, left panel) added to the translation system caused a considerable decrease in binding of the 4F-group initiation factors even to the capped *luciferase* mRNA containing 5′ UTR from *β-globin* mRNA (1D, right panel). Nevertheless, translation of this mRNA was not at all inhibited. Probably, the reason for the minor effect of 4E-BP1 was that the initially high proportion of non-phosphorylated 4E-BP in Krebs extract could hamper further inhibition (Fig. 1D, right panel, lane 1).

**Figure 2:**
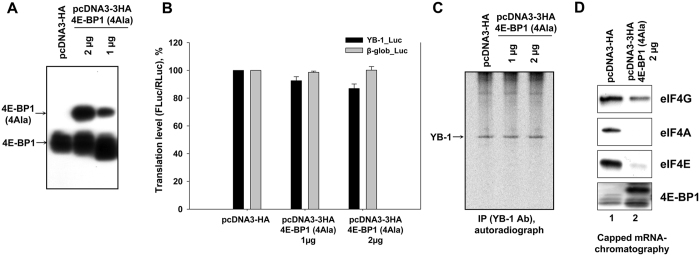
The effect of 4E-BP(4Ala) on translation of reporter mRNAs and on YB-1 synthesis in HeLa cells. (**A**) 10^5^ HeLa cells were transfected by 1 or 2 μg pcDNA3-3HA-4E-BP1(4Ala) or 2 μg pcDNA3-HA plasmids, cultivated for 36 h and used for Western-blot analysis with anti-4E-BP1 antibody. (**B**) 4E-BP1(4Ala)-overexpressing or control HeLa cells (10^5^ each) were transfected by reporter *Firefly luciferase* mRNAs with *YB-1* mRNA- or *globin* mRNA 5′ UTR and *Renilla luciferase* mRNA (as internal control), cultivated for 2 h, harvested and assayed for *Firefly* and *Renilla* luciferase (*FLuc* and *RLuc*, respectively). The *FLuc*/*RLuc* ratio for the control (untreated cells) was taken as 100%. Values are the means of at least three independent experiments. Errors are 2 standard deviations. (**C**) 4E-BP1(4Ala)-overexpressing or control HeLa cells (10^6^ each) were labeled with [^35^S]-methionine for 2 h, harvested and lysed. Cell lysates were used for immunoprecipitation with anti-YB-1 antibody. Proteins bound to antibodies were resolved by acid-urea PAGE, and [^35^S]-labeled proteins were detected by autoradiography. (**D**) Biotinylated, capped *luciferase* mRNA with *YB-1* mRNA 5′ UTR (0.32 pmol) was incubated in 150 μl lysates of 4EBP(4Ala)-overxpressing HeLa cells (2 × 10^5^) or control HeLa cells (2 × 10^5^) and immobilized on Streptavidin-Sepharose. RNA-bound proteins were eluted, separated by SDS-PAGE and analyzed by Western blotting.

Next, we used living cells as a more adequate system to learn the effect of 4E-BP1 on translation of reporter mRNAs. In experiment, the mutant non-phosphorylatable 4E-BP1 (4E-BP1(4Ala)) overexpressed in HeLa cells (Fig. 2A) failed to induce either any significant inhibition of translation of *luciferase* mRNA carrying the 5′ UTR of *YB-1* mRNA (Fig. 2B) or inhibition of synthesis of endogenous YB-1 (Fig. 2C).

Then, we addressed the issue of activity of the cell-expressed protein 4E-BP1(4Ala). In experiment, *luciferase* mRNA carrying *YB-1* mRNA 5′ UTR was biotinylated, capped, and incubated with control HeLa cell lysate or lysate of 4E-BP1(4Ala)-overexpressing HeLa cells using the same buffer as for the cell-free translation system. After immobilization of biotinylated RNA on Streptavidin-Sepharose, RNA-bound proteins were eluted and analyzed by Western blotting to detect translation initiation factors 4E, 4G, 4A, and 4E-BP1. It is seen that 4E-BP1(4Ala) effectively binds to the capped biotinylated reporter mRNA carrying *YB-1* mRNA 5′ UTR (Fig. 2D). This binding is rather caused by interaction of 4E-BP1(4Ala) with eIF4E retained by the cap of this mRNA and prevents interaction between eIF4E and eIF4G-eIF4A (Fig. 2D).

So, it is doubtful whether under mTOR inhibition the suppression of *YB-1* mRNA translation is mediated solely by the 4E-binding protein. Probably, apart from the presence of hypophosphorylated 4E-BP1, efficient inhibition of translation requires an additional mTOR inhibition-related event.

### Interactions of the eIF4F-group factors with 5′ UTRs of various mRNAs in conditions of mTOR inhibition

**Figure 3:**
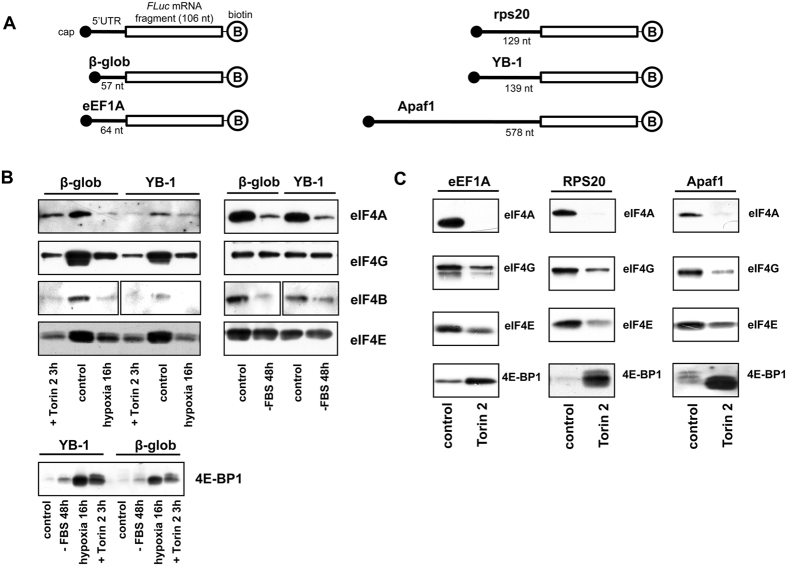
Binding of translation initiation factors to 5′ UTRs of various mRNAs in HeLa cell lysates obtained under mTOR inhibition, serum starvation, or hypoxia. (**A**) A scheme of fragments used in experiment. Fragments with indicated 5′ UTRs and a 106 nt sequence of the luciferase mRNA-encoding region were 3′-biotinylated and 5′-capped. (**B**,**C**) The biotinylated, capped RNA fragments (2 pmol each) were incubated with 150 μl lysates of Torin2-treated, serum starved, exposed to hypoxia or untreated HeLa cells and immobilized on Streptavidin-Sepharose. RNA-bound proteins were eluted, separated by SDS-PAGE, transferred onto a nitrocellulose membrane, and detected using appropriate antibodies.

To elucidate the mechanism of specific *YB-1* mRNA translation inhibition under the effect of mTOR kinase inhibition, we monitored binding of translation initiation factors to the 5′-terminal sequence of reporter *luciferase* mRNA carrying 5′ UTRs of various mRNAs (Fig. 3A). In experiment, lysates of the following HeLa cells were used: untreated cells (control), cells pre-treated with the mTOR inhibitor Torin2, cells subjected to 48 h serum starvation, and cells subjected to hypoxia (1% O_2_, 16 h); the effect of the last two treatments is believed to be mediated mostly by the mTOR signaling pathway^4^. As seen from Fig. 3B, in control, eIF4A, eIF4B, eIF4E and eIF4G appeared bound to all the capped 5′ UTR-carrying fragments, while 4E-BP1, as it was expected, showed virtually no interaction with this RNA. In lysate of Torin2-treated cells, the binding of translation initiation factors was decreased, with drastically increasing binding of the 4E-binding protein. Thus, mTOR inhibition was accompanied by a lower level of eIF4E binding to capped mRNA and a higher level of 4E-BP1 binding to eIF4E retained by the cap-structure, which most probably prevented eIF4E binding to eIF4G. The decrease in eIF4A binding might either result from reduced eIF4G binding to mRNA or probably occur by the PDCD4-mediated mechanism^9^,^10^. In the latter case, the PDCD4-eIF4A interaction may prevent eIF4A binding to eIF4G still bound to mRNA.

**Figure 4:**
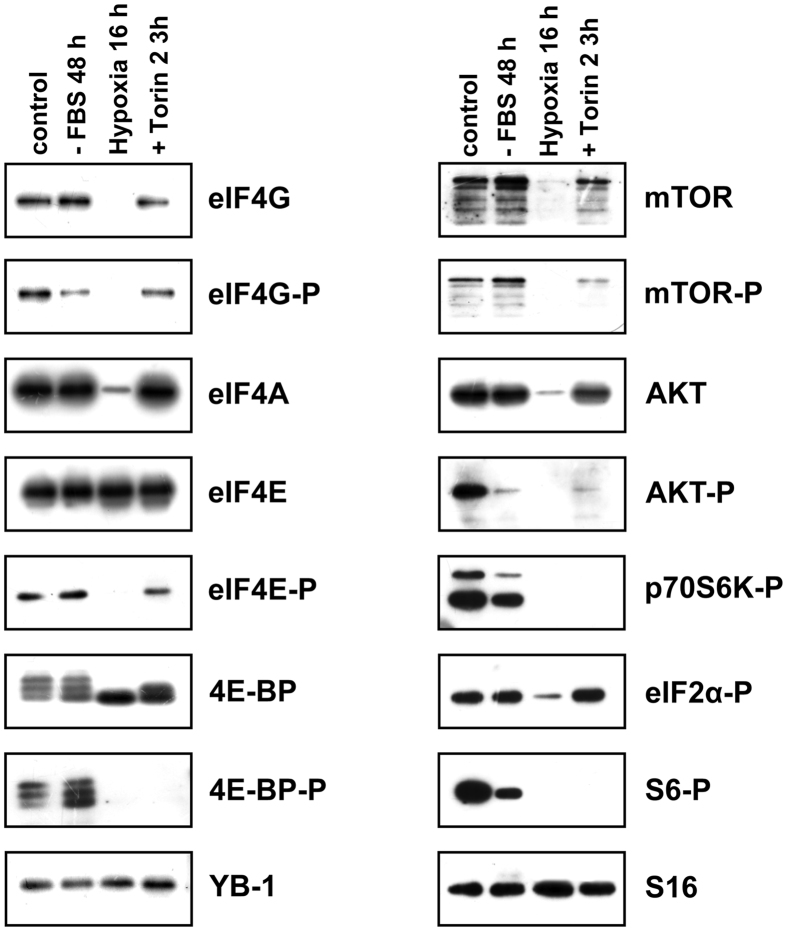
The effect of various treatments on the amount and phosphorylation of key proteins involved in mTOR-mediated translation regulation. Untreated, Torin2-treated, serum starved or exposed to hypoxia HeLa cells were harvested, lysed and used for Western-blotting.

Experiments on binding of the same proteins from hypoxia-subjected cells to capped fragments of *YB-1* mRNA and *β-glob* mRNA demonstrated the same changes as in the case of mTOR inhibition with Torin2. However, the absence of eIF4A and eIF4G binding observed here can be explained not by their lessened affinity for RNA but by a considerable decrease in their amount after 16 h hypoxia (Fig. 4).

The results of the experiment on cell lysate subjected to serum starvation could be expected to be similar to those for mTOR inhibition with Torin2, because as believed, the absence of growth factors eventually affects the mTOR signaling pathway. Nevertheless, no dramatic changes in binding of eIF4E, eIF4G, and 4E-BP1 were observed, but the level of eIF4A and eIF4B binding was much lower. This may indicate that serum starvation influences translation not merely through the 4E-BP-affecting part of the mTOR signaling pathway.

It was of particular importance to us that binding modulations of the eIF4F-group factors and 4E-BP1 appeared to be the same both for mRNAs with translation suppressed upon mTOR inhibition (*YB-1*- and *eEF1A* mRNAs) and those with translation almost independent of mTOR inhibition (*β-glob*-, *rps20*-, *Apaf1* mRNAs). This suggests that specific inhibition of translation of *YB-1* mRNA and some other mRNAs observed upon inhibition of the mTOR signaling pathway resulted not from specifically altered affinity of initiation factors but probably from a stronger dependence of *YB-1* mRNA translation on the eIF4F-group factors. However, since in the absence of inhibition of reporter mRNA translation the overexpression of non-phosphorylatable 4E-BP1 led to decreased binding of the eIF4F-group initiation factors, it is of importance that Torin2-induced mTOR inhibition caused not only inactivation of the eIF4F-group factors but also that of other proteins responsible for cap-binding and RNA-helicase activities during the cap-dependent translation.

### The effect of inhibition of cap-binding and RNA-helicase activities on translation of reporter mRNAs carrying various 5′ UTRs

It was hypothesized that mTOR inhibition has a more dramatic effect on mRNAs whose translation shows a stronger dependence on initiation factors mediating the cap-dependent translational mechanism. To verify this supposition, we checked up whether translation of the used reporter mRNAs in Krebs extracts was affected by the following inhibitors of cap-dependent translation: the cap-analog (m^7^GpppG), and in particular, helicase 4A inhibitors hippuristanol and dominant-negative eIF4A mutant R362Q (eIF4A (R362Q)). Hippuristanol is a small molecule which mostly modulate the eIF4A activity^11^, while eIF4A (R362Q) inhibits the eIF-4F-mediated RNA helicase activity^12^. Suppression of eIF4A activity was of special interest, because binding of this factor to the mRNA fragments invariably altered, no matter to what treatment the HeLa cells were subjected in our experiments. The mRNAs whose translation was suppressed upon mTOR inhibition were expected to be more sensitive to inhibition of cap-dependent translation by all mechanisms used in this study.

**Figure 5:**
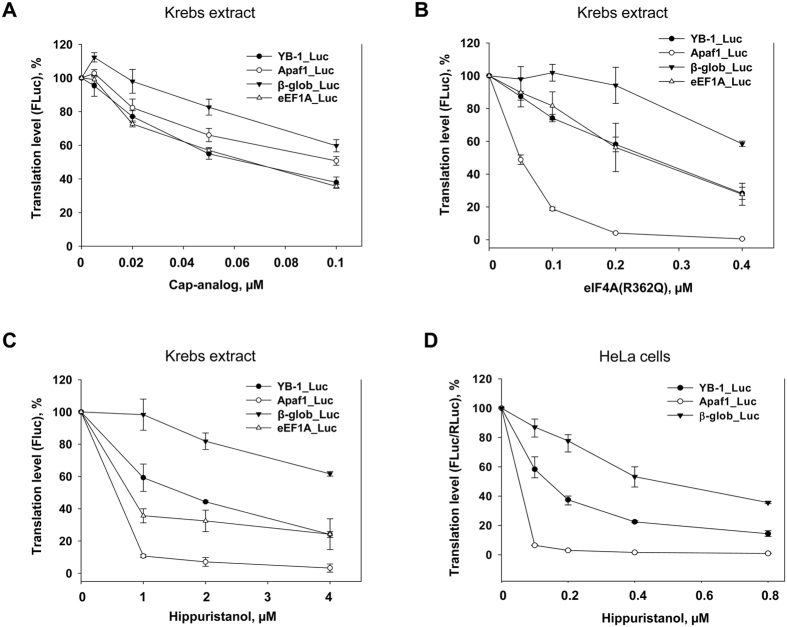
The effect of cap-analog, eIF4A(R362Q), and hippuristanol on translation of reporter mRNAs *in vitro* and in HeLa cells. (**A**–**C**) 0.1 pmol C^+^A^+^ reporter *Firefly luciferase* mRNAs with indicated 5′ UTRs were translated in Krebs extract in the presence of increasing concentrations of cap-analog (0.005, 0.02, 0.05, and 0.1 mM) or without it (**A**), or recombinant eIF4A(R362Q) (0.05, 0.1, 0.2 and 0.4 nmol/ml) or without it (**B**), or hippuristanol (1, 2, and 4 μM) or without it (**C**). Reaction mixtures were assayed for *Firefly* luciferase after 45 min incubation at 30 °C. The *FLuc* activity without addition of cap-analog (**A**) or eIF4A(R362Q) (**B**), or hippuristanol (**C**) was taken to be 100%. Values are the means of at least three independent experiments. Errors are 2 standard deviations. (**D**) Untreated or treated with hippuristanol during 1 h (0.1, 0.2, 0.4, and 0.8 μM) HeLa cells were transfected by reporter *Firefly luciferase* mRNAs with indicated 5′ UTRs and *Renilla luciferase* mRNA (as internal control), cultivated for 2 h, harvested and assayed for *Firefly* and *Renilla* luciferase (*FLuc* and *RLuc*, respectively). The *FLuc*/*RLuc* ratio for the control (untreated cells) was taken as 100%. Values are the means of at least three independent experiments. Errors are 2 standard deviations.

As seen from Fig. 5A, over the indicated concentration range, the reporter RNAs carrying 5′ UTRs from *YB-1*- and *eEF1A* mRNAs whose translation was potently suppressed by Torin2 appeared to be more sensitive to the cap-analog, while translation of *luciferase* mRNA carrying 5′ UTR from *β-globin* mRNA was inhibited to a lower degree.

**Figure 6:**
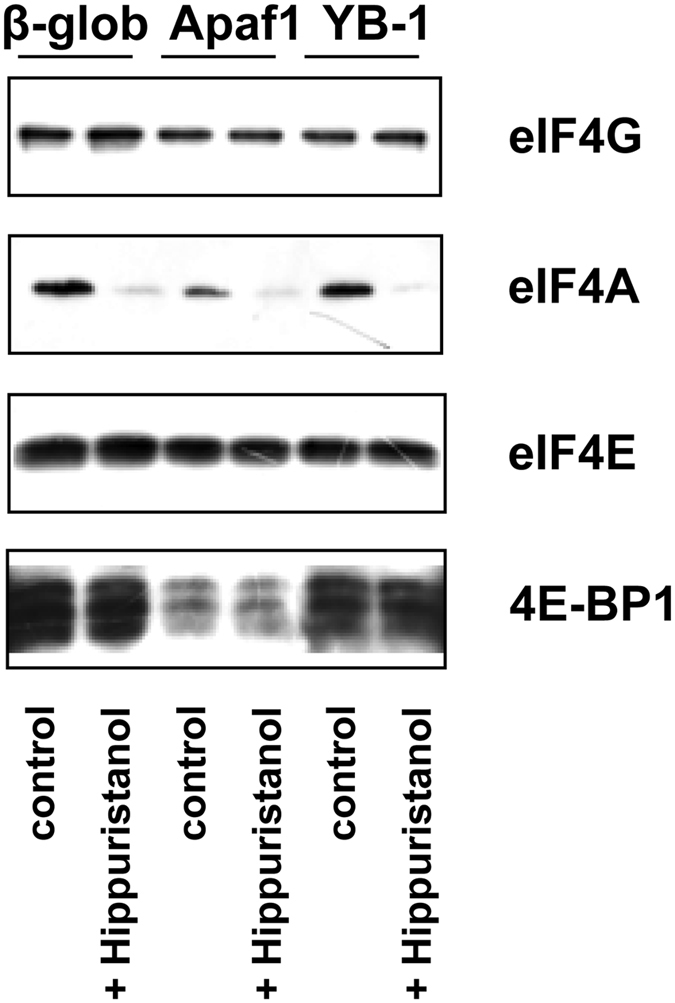
Binding of translation initiation factors to 5′ UTRs of various mRNAs in hippuristanol-treated cells. The biotinylated, capped RNA fragments (2 pmol each) were incubated with 150 μl lysates of hippuristanol-treated or untreated HeLa cells and immobilized on Streptavidin-Sepharose. RNA-bound proteins were eluted, separated by SDS-PAGE, transferred onto a nitrocellulose membrane, and detected using appropriate antibodies.

For Krebs extract, inhibition of the RNA-helicase activity by mutant eIF4A (R362Q) or by hippuristanol showed similar results, except for 5′ UTR from *Apaf1* mRNA. Again, translation of reporter mRNAs with 5′ UTRs from *YB-1*- and *eEF1A* mRNAs was inhibited stronger than that of *luciferase* mRNA with 5′ UTR from *β-globin* mRNA (Fig. 5B). Close results were shown by translation of the reporter mRNAs in HeLa cells in the presence of hippuristanol (Fig. 5D). Importantly, in the case of hippuristanol-treated HeLa cells, eIF4A was the only studied factor that showed a decreased level of binding to the mRNA 5′-terminal sequence (Fig. 6). Thus, our results demonstrate a good concordance with the supposition that *YB-1* mRNA translation is strongly dependent on cap-binding and RNA-helicase activities.

Interestingly, the mRNA with *Apaf1* mRNA 5′ UTR resistant to mTOR kinase inhibitors displayed high sensitivity to both addition of eIF4A (R362Q) and hippuristanol to Krebs extract and addition of hippuristanol to HeLa cells. This is hardly surprising, because, as shown, this mRNA has a long structured 5′ UTR and is translated by the 5′-end-dependent scanning mechanism^13^, and hence, strongly requires a eIF4A-like RNA helicase. Besides, translation of *luciferase* mRNA carrying the 5′ UTR from *Apaf1* mRNA was also inhibited by the cap-analog, though to a lower degree than in the case of *luciferase* mRNA carrying the *YB-1* mRNA 5′ UTR. However, in spite of a decreased level of eIF4A and eIF4E binding to *Apaf1* mRNA 5′ UTR (Fig. 3C), translation of *luciferase* mRNA carrying the same leader was not suppressed in HeLa cells upon mTOR inhibition (Fig. 1B). For plausible interpretation, see Discussion.

### The effect of hippuristanol on YB-1 synthesis *in vivo*

**Figure 7:**
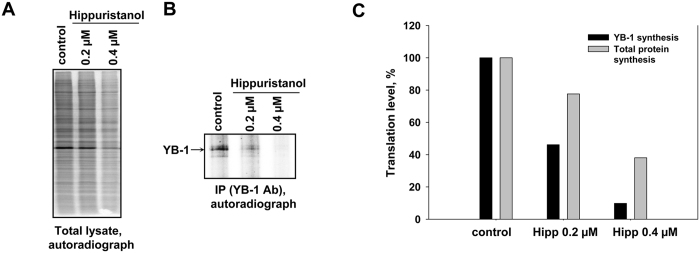
The effect of hippuristanol on total protein synthesis and YB-1 synthesis in HeLa cells. HeLa cells were treated with hippuristanol (0.2 and 0.4 μM) for 1 h. Untreated and hippuristanol-treated cells were labeled with [^35^S]-methionine for 2 h, harvested and lysed. Cell lysates were counterbalanced by the total protein amount and used for SDS-PAGE for total protein synthesis quantification (**A**) or for immunoprecipitation with anti-YB-1 antibodies. Proteins bound to antibodies were resolved by acid-urea PAGE, and [^35^S]-labeled proteins were detected by autoradiography (**B**). Relative radioactivity of the bands or lanes was determined using a Packard Cyclone Storage Phosphor System (Packard Instrument Company, Inc.). The level of total protein synthesis or YB-1 synthesis in hippuristanol-untreated cells was taken to be 100% (**C**).

It is of interest that the recent paper by Rubio *et al*.^14^ did not mention *YB-1* mRNA among mRNAs with translation sensitive to silvestrol, another inhibitor of eIF4A. For additional verification of the fact that YB-1 synthesis is sensitive to inhibition of the eIF4A activity, we studied the effect of hippuristanol on synthesis of endogenous YB-1. As seen from Fig. 7, in HeLa cells, YB-1 synthesis was inhibited much stronger than the total protein synthesis.

Thus, we demonstrate that translation of *YB-1* mRNA shows a higher sensitivity to inhibition of the canonical cap-dependent scanning mechanism of protein synthesis initiation; probably, this hypersensitivity underlies a stronger dependence of translation of some mRNAs, including *YB-1* mRNA, on the level of activity of the mTOR signaling pathway.

## Discussion

Initially, this study was focused on the mechanism of mTOR-dependent regulation of *YB-1* mRNA translation. However, the obtained results allow a broader conclusion applicable to other mRNAs as well. Our initial supposition was that 4E-BP is the key protein in regulation of *YB-1* mRNA translation by the mTOR signaling pathway. We showed that overexpression of 4E-BP1 in HeLa cells or its addition to a cell-free translation system resulted in decreased binding of the eIF4F-group initiation factors to reporter mRNAs carrying 5′ UTRs from YB-1 mRNA, but this gave no significant inhibition of translation.

Meanwhile, translation of *YB-1* mRNA was specifically and strongly inhibited by the mTOR inhibitor Torin2. Besides, we showed that *YB-1* mRNA and other mRNAs, both sensitive and nonsensitive to mTOR inhibition, demonstrated an equal significant decrease in their binding to eIF4G, eIF4E, and eIF4A in particular, with increasing binding to 4E-BP. Together, these data have shown that 4E-BP1 alone (allegedly the major target for translation regulation by the mTOR signaling pathway) is insufficient for potent suppression of mRNA translation; presumably, mTOR inhibitors additionally influence the translation initiation mechanisms, thereby promoting specific inhibition of some mRNAs.

Supposedly, this additional effect occurring upon mTOR inhibition consists in 4E-BP-independent inhibition of the cap-binding and RNA-helicase activities of factors involved in translation initiation, including the canonical initiation factors eIF4E and eIF4A. The role of 4A is not a novel finding, since the protein PDCD4, a substrate of this pathway, is shown to act as an inhibitor of eIF4A activity^9^,^10^. The same is true for the role of eIF4B that is phosphorylated by S6-kinase, which results in its higher affinity for eIF3; this may affect translation initiation^15^. It is of interest that cell treatment with the mTOR kinase inhibitor Torin2 makes lower the eIF4E binding itself, which has not been reported so far because similar experiments commonly use cap-Sepharose^6^,^16^, unlike our study using fragments of capped mRNA. Probably, the decreased level of the eIF4E-to-mRNA binding may be partially explained by a lower affinity for the capped RNA caused by the broken contact between eIF4G and eIF4E. However, it cannot be ruled out that mTOR inhibition not only impedes the contact between eIF4E and eIF4G due to binding of the latter to non-phosphorylated 4E-BP but also decreases the level of eIF4E binding to mRNAs. The reason for the phenomenon is still obscure.

Our supposition is that specific inhibition of mRNA translation upon suppression of the mTOR signaling pathway is underlain not by specifically altered affinity of initiation factors but probably by a stronger dependence of mRNA translation on activity of the eIF4F-group factors or other proteins of similar activities. Specifically for *YB-1* mRNA, we showed that its translation is only slightly resistant to inhibition of activity of translation initiation factors by the cap-analog (mutant eIF4A (R362Q)) and by hippuristanol. The reason for such a behavior is unclear, and it possibly differs from mRNA to mRNA or from one small group of mRNAs to another; it may result from peculiarities of the secondary structure or binding of certain proteins to a certain sequence within the 5′ UTR, or something else. Probably this explains the diversity of proteins interacting with the TOP-motif (La, LARP7, LARP1, CNBP/ZNF9, AUF1, TIAR/TIA1) and allegedly participating in translation regulation^8^. However, experiments on knockout of these proteins did not confirm their key role in mRNA translation regulation mediated by the mTOR signaling pathway^8^,^17^.

Besides, it seems most probable that among mRNAs sensitive to mTOR inhibition there are those highly sensitive to suppression of the eIF4E activity (possibly, the largest group) or to inhibition of eIF4A activity, and those depending on both eIF4E- and eIF4A activities. This categorization may explain a distinct division of all mRNAs into two groups: those with translation suppressed by the mTOR inhibitor INK128 and those with translation inhibited by silvestrol, an inhibitor of the eIF4A activity^14^. The latter group contains small percentage of TOP and TOP-like mRNAs that may be sensitive to mTOR inhibition^14^. According to our results, *YB-1* mRNA may be classified with mRNAs sensitive to both cap-binding and RNA-helicase activities.

Our data on the *Apaf1* mRNA 5′ UTR are of significance. This mRNA is extremely sensitive to eIF4A activity inhibition both *in vitro* and *in vivo*, but its translation remains unsuppressed by mTOR inhibition implying a decreased level of mRNA-to-eIF4A binding. This may be explained by the fact that the first step in mTOR inhibition is shutdown of the canonical scanning mechanism involving eIF4A, probably without inhibition of other RNA helicases that replace eIF4A in *Apaf1* mRNA 5′ UTR scanning. Together with a somewhat lower sensitivity of *Apaf1* mRNA to the decreasing amount of accessible eIF4E, this replacement allows translation in conditions of inhibited mTOR. However, in the case of incubation of these cells with hippuristanol, that presumably inhibited not only 4A but also other helicases required for scanning the *Apaf1* mRNA 5′ UTR, translation of this mRNA was suppressed. The fact that in Krebs extract translation of mRNA with the *Apaf1* mRNA 5′ UTR was inhibited by addition of eIF4A (R362Q) may be explained by the dual activity of this mutant: it supported affinity for eIF4G but prevented interaction of eIF4G with other helicases. Also, it is worth noting a significant decrease of 4E-BP1 binding to Apaf1 5′ UTR, as compared with β-globin and YB-1 UTRs (Fig. 6). Our supposition is that in these experiments 4E-BP1 binding could occur only as a result of its interaction with eIF4E. But for all that, only non-phosphorylated 4E-BP underwent binding. At the same time, the amount of bound eIF4E was the same for all used RNAs, which shows that in the case of Apaf1 5′ UTR the 4E-BP binding was hampered by something. This “something” is difficult to be defined. Perhaps it may be the secondary structure of Apaf1 5′ UTR or interaction of this leader with other proteins interfering with contacts between eIF4E and 4E-BP.

Supposedly, mRNAs capable of using alternative mechanisms of translation initiation that involve analogs of canonical translation initiation factors are also capable of being well translated in conditions of inhibited mTOR, provided the analogs retain their activities under these conditions. Hence, the problem of mTOR-dependent regulation should be approached otherwise. It demands disclosure and investigation of not only mechanisms responsible for specific inhibition of translation of TOP- and TOP-like mRNAs but also those providing continuous translation of some other mRNAs under conditions of mTOR inhibition. A lower dependence of some mRNAs on the translation initiation factors and the use of proteins capable of performing the functions of canonical eIF4s may serve as such alternative mechanisms. Primarily, these are RNA helicases with an activity analogous to that of eIF4A, namely, DHX29, Ded1/DDX3, Vasa/DDX4, RNA helicase A (RHA/DHX9) and Dhh1/RCK/DDX6^18^. Secondly, there can be mentioned eIF4G-like proteins, for example, eIF4G2(p97/DAP5/NAT-1)^19^ and Mextli (Mxt) in an invertebrate^20^, or other cap-binding proteins, such as 4EHP^21^.

Interestingly, the helicase eIF4A appeared to be the only protein with a lower level of binding to capped mRNA under serum (growth factors) starvation conditions. This not only points to the importance of the eIF4А activity in regulation of translation with deficient growth factors but also emphasizes the difference between mTOR inhibitor-involving and uninvolving mechanisms of translation regulation. Lately, this hypothesis gains more and more evidence^8^.

## Materials and Methods

### Plasmid construction

The pSP36T-5′ UTR YB1-FLuc-3′ UTR GAPDH-A50 and pSP36T-5′ UTR β*-*globin-FLuc-3′ UTR GAPDH-A50 were obtained as described previously^3^.

The 5′ UTRs of Apaf1 and rps20 mRNAs were obtained by RT-PCR amplification of total RNA of HeLa cells. The forward primer for Apaf1 5′ UTR was 5′-AT**AAGCTT**AAGAAGAGGTAGCGAGTGGACGT-3′, the reverse primer was 5′-AT**CCATGG**TCCCTCAGATCTTTCTC-3′ (the *HindIII* and *NcoI* restriction sites are bold). The forward primer for rps20 5′ UTR was 5′-TG**AAGCTT**CCTTTCTTTTTGAGGAAGAC-3′, the reverse primer was 5′-TG**CCATGG**CTGTTGCGCGCGGGCTT-3′ (the *HindIII* and *NcoI* restriction sites are bold). The PCR products were treated with *HindIII* and *NcoI* and ligated into pSP36T-5′ UTR β-globin-FLuc-3′ UTR GAPDH-A50 treated with the same restriction endonucleases. The constructs were named pSP36T-5′ UTR Apaf1-FLuc-3′ UTR GAPDH-A50 and pSP36T-5′ UTR rps20-FLuc-3′ UTR GAPDH-A50.

The 5′ UTR of EEF1A mRNA was obtained by annealing two oligonucleotides (5′-**AGCT**TTTTCGCAACGGGTTTGCCGCCAGAACACAGGTGTCGTGAAAACTACCCCTAAAAGCCAAAC-3′ and 5′-**CATG**GTTTGGCTTTTAGGGGTAGTTTTCACGACACCTGTGTTCTGGCGGCAAACCCGTTGCGAAAA-3′; sticky ends similar to those formed after *HindIII* and *NcoI* restriction are shown in bold) followed by their ligation into pSP36T-5′ UTR β-globin-FLuc-3′ UTR GAPDHA50 treated with *NcoI* and *HindIII* restriction endonucleases. The construct was named pSP36T-5′ UTR EEF1A-FLuc-3′ UTR GAPDH-A50.

pGL3–5′ UTR GADD45-Luc was kindly provided by Dr. Ilya Terenin.

DNA template for *RLuc* RNA was obtained by PCR amplification of the pGL3-RLuc plasmid^22^ with the T7 promoter-containing forward primer 5′-CGCCGTAATACGACTCACTATAGGGTACAAGCTTACCATGACTTCGAAAGTTTATGATCCAG-3′ and the reverse primer 5′-(T50)AACTTGTTTATTGCAGCTTATAATGG -3′.

DNA templates for RNA fragments used in protein purification experiments were obtained by PCR amplification of the corresponding plasmid with the SP6 promoter forward primer 5′-ATTTAGGTGACACTATAG-3′ and the reverse primer 5′-CCAGGGCGTATCTCTTCATAGCCT-3′ complementary to the sequence of luciferase cDNA (83–106 nt).

### *In vitro* transcription

*Firefly Luciferase* (*FLuc*) cap^−^poly(A)^+^ mRNA with *YB-1* mRNA 5′ UTR and *GAPDH* mRNA 3′ UTR was transcribed by SP6 RNA polymerase from pSP36T-5′ UTR YB1-FLuc-3′ UTR GAPDH-A50 linearized with *HpaI. Firefly Luciferase* (*FLuc*) cap^−^poly(A)^+^ mRNA with *β-globin* mRNA 5′ UTR and *GAPDH* mRNA 3′ UTR was transcribed by SP6 RNA polymerase from pSP36T-5′ UTR β-globin-FLuc-3′ UTR GAPDH*-*A50 linearized with *HpaI. Firefly Luciferase* (*FLuc*) cap^−^poly(A)^+^ mRNA with *Apaf1* mRNA 5′ UTR and *GAPDH* mRNA 3′ UTR was transcribed by SP6 RNA polymerase from pSP36T-5′ UTR Apaf1-FLuc-3′ UTR GAPDH*-*A50 linearized with *HpaI. Firefly Luciferase* (*FLuc*) cap^−^poly(A)^+^ mRNA with *EEF1A* mRNA 5′ UTR and *GAPDH* mRNA 3′ UTR was transcribed by SP6 RNA polymerase from pSP36T-5′ UTR EEF1A-FLuc-3′ UTR GAPDH*-*A50 linearized with *HpaI. Firefly Luciferase* (*FLuc*) cap^−^poly(A)^+^ mRNA with *rps20* mRNA 5′ UTR and *GAPDH* mRNA 3′ UTR was transcribed by SP6 RNA polymerase from pSP36T-5′ UTR rps20-FLuc-3′ UTR GAPDH*-*A50 linearized with *HpaI*. These reporter mRNAs had the same additional 12 nt sequence upstream of 5′ UTR (see Supplementary Fig. S1).

*Firefly Luciferase* (*FLuc*) cap^−^poly(A)^+^ mRNA with *GADD45* mRNA 5′ UTR was transcribed by T7 RNA polymerase from pGL3–5′ UTR GADD45-Luc linearized with *XbaI*.

*Renilla luciferase* (*RLuc*) mRNA was transcribed by T7 RNA polymerase from corresponding PCR product.

RNA fragments used in protein purification experiments were transcribed by SP6 RNA polymerase from corresponding PCR products and contained 5′ UTRs of various mRNAs and a 106 nt sequence of the *luciferase* mRNA coding region.

Transcription was performed as described previously^23^. Capped mRNA transcripts were obtained using a ScriptCap™ m^7^G Capping System and ScriptCap 2′-O-Methyltransferase Enzyme (CellScript) according to the suppliers’ recommendations.

### Cell cultures, transient transfections, and metabolic labeling

HeLa cells were cultivated in DMEM/F12 supplemented with 10% fetal bovine serum, 2 mM glutamine, 100 U/ml penicillin and 100 μg streptomycin (PanEco). Cells were kept at 37 °C in a humidified atmosphere containing 5% CO_2_. Cells were passaged by standard methods.

Сells were treated with Torin2 (Tocris), or Hippuristanol (kindly provided by Jerry Pelletier, McGill University, Montreal, Canada) for 1 h before RNA transfection. To prepare lysates for affinity chromatography using mRNA fragments immobilized on Streptavidin-Sepharose, HeLa cells (two 100-mm dishes per experiment) were cultivated either with Torin2 (250 nM) for 3 h or with Hippuristanol (2 and 4 μM) for 3 h, or in DMEM/F12 without fetal bovine serum for 48 h, or under hypoxia (1% O_2_ by N_2_ displacement, 5% CO_2_) in a multigas incubator (Galaxy 14S, New Brunswick Scientific) for 16 h. After treatment cells were washed with phosphate buffered saline and lysed in hypotonic buffer (10 mM Hepes-KOH pH 7.6, 30 mM KCl, 0.5 mM MgCl_2_, 1 mM DTT).

For RNA transfection experiments, HeLa cells were plated onto a 24-well dish 24 h prior to transfection of reporter luciferase mRNAs. Cells were transfected using Lipofectamine 2000 (Invitrogen). For a typical RNA transfection, 0.5 μg of FLuc mRNA and 0.05 μg of RLuc mRNA was incubated with 2 μl of the transfection reagent in 100 μl DMEM for 20 min and then added to the growth medium. 2 h later cells were harvested, and luciferase activities were analyzed using a Dual Glo Luciferase Assay kit (Promega). All transfections were repeated several times in different cell passages.

For DNA transfection experiments, HeLa cells were plated onto a 24-well dish 24 h prior to transfection of DNA. Cells were transfected using Lipofectamine 2000 (Invitrogen). For transfection, 1 or 2 μg of the appropriate plasmid (pcDNA3-3HA-h4E-BP1-4Ala^24^ or pcDNA3HA) was incubated with 2 μl of the transfection reagent in 100 μl DMEM for 20 min and then added to the growth medium. 12 hours later, cells were plated onto a new 24-well dish and cultivated for 24 h prior to transfection of RNA or were plated onto a 6-well dish for [^35^S]-methionine labeling experiment.

For [^35^S]-methionine labeling, cells were cultivated with the Dulbecco modified Eagle’s medium without L-methionine but with 0.1 mCi/ml of L-[^35^S]-methionine (Perkin Elmer, 1,000 Ci/mmol) for 3 h. These cells were utilized for immunoprecipitation.

### Translation in Krebs extracts

Translation experiments were performed in a total volume of 10 μl which contained 5 μl of the Krebs (ascites carcinoma cells) S30 extract (kindly provided by Prof. Ivan Shatsky’s team), 1 μl 10X translation buffer (200 mM Hepes–KOH pH 7.6, 10 mM DTT, 5 mM spermidine–HCl, 80 mM creatine phosphate, 10 mM ATP, 2 mM GTP, and 250 μM of each amino acid), 100 mM KAc, 1 mM Mg(Ac)_2_, 2 units of Human Placental Ribonuclease Inhibitor (Fermentas), 0.15 pmol FLuc mRNA, and 0.015 pmol RLuc mRNA for 1 h. The luciferase activities were measured using a Dual Luciferase Assay kit. When indicated, various amounts of m^7^GpppG (equilibrated at each experimental point with a respective amount of ApppG up to 0.1 μM) were added, and the translation mix was pre-incubated for 5 min before addition of mRNA. For experiments with 4E-BP1, the recombinant protein was used. Plasmid pGEX-6p1-h4E-BP1 (kindly provided by Y. Svitkin and N. Sonenberg, McGill University, Montreal) encoding human 4E-BP1-GST fusion was expressed in *Escherichia coli*, and 4E-BP1 was purified using Glutathione-Sepharose 4B and PreScission Protease (Amersham) and dialyzed against buffer A (20 mM Hepes-KOH, pH 7.6, 100 mM KCl, 1 mM DTT, 5% glycerol). Purified 4E-BP1 was added to the translation mix in a volume less than 1.5 μl and incubated for 5 min before addition of reporter mRNAs. In control, an equal amount of buffer A was added.

For experiments with eIF4A(R362Q), the recombinant protein was used. Plasmid pET(His6-R362Q)^25^ encoding human eIF4A(R362Q) was expressed in *Escherichia coli*, and eIF4A(R362Q) was purified using TALON® Metal Affinity Resin (Clontech) and dialyzed against buffer A (20 mM Hepes-KOH, pH 7.6, 100 mM KCl, 1 mM DTT, 5% glycerol). Purified eIF4A (R362Q) was added to the translation mix in a volume less than 1.5 μl and incubated for 5 min before addition of reporter mRNAs. In control, an equal amount of buffer A was added.

### Western blotting

Proteins were separated by SDS-PAGE and transferred to a nitrocellulose membrane. The membrane was blocked for 1 h at RT with 5% milk and incubated overnight in TBS-T (10 mM Tris-HCl, pH 7.6, and 150 mM NaCl, 0.05% Tween 20) supplemented with BSA (5%) and with appropriate antibodies (polyclonal rat antibody against 14 a.a. C-terminal peptide of YB-1 (IMTEK, Russia), polyclonal rabbit antibody against phospho-mTOR^S2448^ (2971S, Cell Signaling), polyclonal rabbit antibody against mTOR (2972S, Cell Signaling), polyclonal rabbit antibody against phospho-AKT^S473^ (9271S, Cell Signaling), polyclonal rabbit antibody against AKT (9272S, Cell Signaling), monoclonal rabbit antibody against phospho-p70S6K^T389^ (9234S, Cell Signaling), monoclonal rabbit antibody against phospho-rpS6^S240/244^ (4858S, Cell Signaling), polyclonal rabbit antibody against 4E-BP1 (9644S, Cell Signaling), monoclonal rabbit antibody against phospho-4E-BP1^T37/46^ (2855S, Cell Signaling), monoclonal rabbit antibody against nonphospho-4E-BP1 (4923S, Cell Signaling), monoclonal rabbit antibody against eIF4E (2067S, Cell Signaling) polyclonal rabbit antibody against phospho-eIF4E^S209^ (9741S, Cell Signaling), monoclonal rabbit antibody against phospho-eIF2α^S51^ (3398S, Cell Signaling), monoclonal rabbit antibody against eIF4G (2469S, Cell Signaling), polyclonal rabbit antibody against phospho-eIF4G^S1108^ (2441S, Cell Signaling), polyclonal rabbit antibody against eIF4A (2013S, Cell Signaling), polyclonal rabbit antibody against eIF4B (3592S, Cell Signaling), polyclonal rabbit antibody against rps16 (ab26159, Abcam)). Immunocomplexes were detected using a ECL kit (GE Healthcare) according to the manufacturer’s recommendation.

### Immunoprecipitation, YB-1 synthesis detection

Cells were washed with phosphate buffered saline, scraped and lysed with buffer containing 20 mM Hepes-KOH, pH 7.6, 100 KCl, 5 mM MgCl_2_, 2 mM DTT, 0.25% Nonidet P-40, 0.2% SDS and protease inhibitors cocktail. Cell debris was removed by centrifugation at 10,000 rpm for 15 min, and extracts were then directly utilized for IP. For IP, cell extracts were incubated with anti-YB-1 antibodies (approximately 100 μg) immobilized on protein G-Sepharose beads (GE Healthcare) for 2 h at 4 °C. After extensive washing with PBS, the proteins were eluted with acid-urea sample buffer (8 M urea, 5% glacial acetic acid, 0.025 methylene blue), and analyzed by acid-urea 10% polyacrylamide gel electrophoresis (AUE) and autoradiography. Relative radioactivity of the bands or lanes was determined using a Packard Cyclone Storage Phosphor System (Packard Instrument Company, Inc.). The level of total protein synthesis or YB-1 synthesis in untreated cells was taken to be 100%.

#### mRNA fragment biotinylation and capping

3′-end biotinylation was performed as described previously^26^. Briefly, a 50 μg RNA sample was oxidized in 100 μl of fresh solution of 5 mM NaIO_4_ in 75 mM NaOAc buffer (pH 4.5). The reaction was performed in the dark at 4 °C for 45 min. After ethanol precipitation, the RNA was dissolved in 100 μl of fresh solution of 10 mM biotin hydrazide (Calbiochem, USA) in dimethyl sulfoxide. Then the samples were incubated for 4 h at 37 °C, and each of them was supplemented with 100 μl of 200 mM NaBH_4_ and 200 μl of 1 M Tris-HCl (pH 8.0) with subsequent incubation in the dark at 4 °C for 30 min. Then the RNA was ethanol-precipitated and dissolved in the required volume of H_2_O.

After 3′-biotinylation RNA fragments were capped using a ScriptCap™ m^7^G Capping System and ScriptCap 2′-O-Methyltransferase Enzyme (CellScript) according to the suppliers’ recommendations.

#### Protein isolation from cell lysates using biotinylated mRNA fragments

Streptavidin-Sepharose (GE Healthcare, USA) was washed five times with 30 volumes of PBS supplemented with up to 0.2 mM VRC (Fluka, Switzerland). Then the resin was incubated overnight with a blocking solution (PBS supplemented with BSA up to 1 mg/ml, glycogen up to 200 μg/ml, and total RNA from *E. coli* up to 200 μg/ml) at 4 °C with constant stirring. After incubation, the resin was washed five times with 10 volumes of PBS and 2 times with 10 volumes of buffer for *in vitro* translation (20 mM Hepes–KOH, pH 7.6, 1 mM DTT, 0.5 mM spermidine–HCl, 1 mM Mg(Ac)_2_, 8 mM creatine phosphate, 1 mM ATP, 0.2 mM GTP, 100 mM KAc, and 25 μM of each amino acid). The 2 pmol capped biotinylated RNA was incubated with 40 μl HeLa cell lysate in *in vitro* translation buffer supplemented with 20 μg of total *E. coli* RNA for 15 min at 30 °C. This mixture was then supplemented with 35 μl of 50% pre-blocked Streptavidin-Sepharose slurry and incubated for 1 h at room temperature. The resin with RNA–protein complexes was washed 8 times with 30 volumes of PBS (with up to 0.2 mM of VRC added). Bound proteins were eluted with the same buffer as for SDS-PAGE (80 mM Tris-HCl, pH 6.8, 2% SDS, 200 mM 2-mercatoethanol, 10% glycerol, and 0.0012% bromophenol blue), separated by 10% SDS-PAGE and analyzed by Western blotting.

## Additional Information

**How to cite this article**: Lyabin, D. N. and Ovchinnikov, L. P. Selective regulation of YB-1 mRNA translation by the mTOR signaling pathway is not mediated by 4E-binding protein. *Sci. Rep.* **6**, 22502; doi: 10.1038/srep22502 (2016).

## Supplementary Material

Supplementary Information
