# Supplementary material for: Selective regulation of YB-1 mRNA translation by the mTOR signaling pathway is not mediated by 4E-binding protein
Source: Sci Rep. 2016 Mar 2;6:22502. doi: 10.1038/srep22502 (PMC4773878; doi:10.1038/srep22502)
Supplement: Supplementary Information [file srep22502-s1.pdf]

Supplementary Information

Selective regulation of YB-1 mRNA translation by the mTOR signaling pathway is not mediated by 4E-binding protein

Lyabin D.N[1]*. and Ovchinnikov L.P[1].

[1]Institute of Protein Research, Russian Academy of Sciences, Pushchino, 142290, Russian Federation

***corresponding author, lyabin@vega.protres.ru**

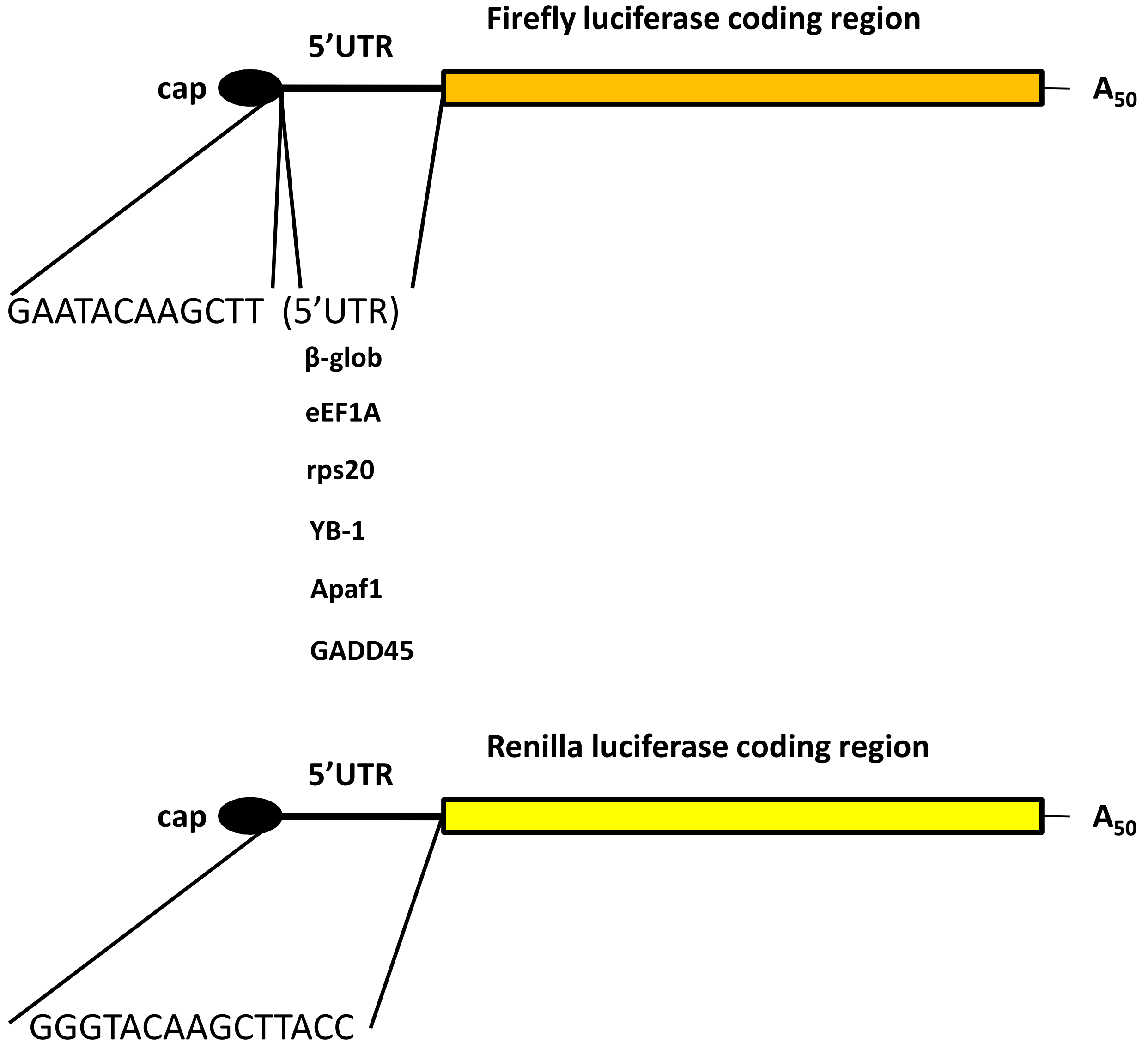

Supplementary figure 1.

A scheme of reporter mRNAs used in experiment.
